# Assessment of the impact of cold plasma technology on physicochemical properties of corn starch flour and the associated modified corn starch incorporated into milk dessert

**DOI:** 10.1016/j.heliyon.2024.e37399

**Published:** 2024-09-03

**Authors:** Hannaneh Bahmanpour, Narmela Asefi, Aynaz Alizadeh, Sajad Pirsa

**Affiliations:** a Department of Food Science and Technology, Tabriz Branch, Islamic Azad University, Tabriz, Iran; b Department of Food Science and Technology, Faculty of Agriculture, Urmia University, Urmia, Iran

**Keywords:** Cold plasma, Corn starch, Milk dessert, Enrichment foods, Physicochemical properties

## Abstract

The utilization of cold plasma can be used as an alternative method to modify the properties of starch. This research aimed to examine the use of cold plasma technology to alter the structure of corn starch and investigate how its functionality could be improved using a food model (milk dessert). Modified corn starch by plasma technology under different gas contents (dielectric-barrier discharge (DBD)) (95 % argon+5 % hydrogen (DBD1) and 90 % argon+10 % oxygen (DBD2)) was compared to the control sample of corn starch. The physicochemical characteristics of modified corn starch, DSC, XRD, SEM and FTIR tests were evaluated. The findings demonstrated that corn starch had significantly higher solubility, transparency, ash, oil absorption capacity (OAC), and resistant starch (RS) when exposed to cold plasma under the test circumstances compared to the control sample. SEM analysis confirmed that plasma affected the surface of starch granules, making the surface changes more pronounced when oxygen was added to the treatment. It was concluded that the sample should be treated with plasma containing 90 % argon and 10 % oxygen (as the best sample). The best sample (modified corn starch) was used to prepare a milk dessert as a food model, and considerable differences were found between the modified starch treated sample and control samples in terms of moisture, brix, syneresis, and organoleptic properties (p < 0.05).

## 1 Introduction

Starch plays an important role in human nutrition and is one of the most important biopolymers used in various fields of the food industry (as a bulking agent and stabilizer), paper, textile, pharmaceutical, etc. [1]. Starch in plants is produced in components called amyloplast. Each amyloplast has one or more or less spherical bodies called starch granules. Actually, the storage place of starch is inside the granules, and the shape and size of the granules are different depending on the type of plant [2]. Native starch is altered to improve the characteristics of its industrial usage [3]. The variation in the properties of starch is principally owing to depolymerization and cross-linking of amylose and amylopectin side chains [4,5].

Various methods can be employed to enhance the properties of starch, including enzymatic, chemical, and physical approaches. While chemical methods boost the efficacy of modified starches, they have drawbacks such as high costs, time consumption, and chemical residues. Therefore, the utilization of physical techniques, which are rapid, non-toxic, and environmentally compatible, has been considered. Radiation, ultrasound waves and cold plasma are the famous physical methods to produce modified starch [6]. Numerous physical, chemical, and enzymatic methods have been used for treating starch. Adopting environmentally responsible physical techniques (i.e., cold plasma) appears more appropriate for increasing the physicochemical properties of products than alternative treatment methods [7].

The use of cold plasma processing as a completely new and non-thermal processing technique is being investigated due to the increasing influence of minimally processed foods [8]. Cold plasma can be formed using different equipment and energy sources such as electricity, heat, and electromagnetic waves [9]. The kind of feed gas, the voltage used, and the length of the treatment determines how cold plasma reacts with natural polymers like starch.

Okyere et al. (2019) used the cold plasma of the CO2-Ar radiofrequency to treat the starch produced from rice and potato [7]. All samples of potato starch treated with plasma and the gas mixture led to a reduced crystallinity. Plasma dielectric discharge changed the surface of starch granules and their internal structures by creating holes in them. Furthermore, after plasma treatment, the relative degree of crystallinity and the molecular weight decreased with increasing hole diameter at the surface of the molecules. After plasma treatment, a reduction in viscosity, molecular weight, and gelatinization temperatures resulted. Furthermore, plasma etching augmented the surface energy, and improved the hydrophilicity of the starch granules [10]. In summary, it can be concluded that cold plasma is an alternative method to modify the properties of starch.

The growth of consumers' attention to the role of nutrition in health and well-being is the first driver for the production of super-beneficial foods. In recent years, the desire to produce and consume ultra-beneficial foods is increasing. Milk and dairy products are an important part of healthy foods. Milk plays an important role in human diet due to its protein, calcium, phosphorus, iron, riboflavin and vitamins A and B12. Milk-based dessert is one of the dairy products that, in addition to its nutritional value, creates variety in the consumer's product basket. The most important feature of milk dessert is its high energy and the pleasant feeling it creates in the consumer. Useful dairy drinks such as milk dessert with improved physicochemical properties and long shelf life have attracted the attention of consumers of dairy products [11].

Considering the high importance of starch modification using cold plasma mentioned above, which improve the physicochemical properties of corn starch, in this study cold plasma technology (with two different combinations of two gases) was used. Also, corn starch treated with cold plasma was used in the preparation of milk dessert as a food model.

## 2 Materials and methods

### 2.1 Materials

Edible corn starch was obtained from Zarnam Industrial and Research Group (Iran-Tehran). Other required chemical compounds were obtained and used from Merck (Germany) and Aldrich (USA).

### 2.2 Plasma treatment of corn starch

For this purpose, 10 g of corn starch were added to the equipment chamber. Plasma irradiation was carried out using three types of gas, involving argon, nitrogen, and oxygen, for 10 min by adjusting the gas current and voltage to 6 kV and a frequency of 16 kHz. The sample was mixed numerous times [9]. In this investigation, samples were treated in the dielectric-barrier discharge (DBD) device. The condition of 95 % argon gas and 5 % hydrogen gas was named as DBD1 and the condition of 90 % argon gas and 10 % oxygen gas was named as DBD2. The untreated corn starch sample was used as control sample [12].

### 2.3 Characterization of corn starch after plasma treatment

#### 2.3.1 Determination of physicochemical characteristics

Measurement of transparency of modified starch gel were determined by Hu et al. (2016) method [13]. To determine the oil absorption capacity (OAC), the method described by de la Hera et al. (2013) was used [14]. The hydration characteristics of treated corn starch samples were investigated by examining the water solubility index (WSI) and swelling power (SP) at different temperatures (55, 65, 75, 85, and 95 °C) [9,15]. To evaluate the digestibility of the treated starch samples under laboratory conditions and compare them with the control sample, the three factors of resistant starch (RS), slowly digestible starch (SDS) and resistant digestible starch (RDS) were measured by Ge et al. (2021) method (with slight modifications) [16].

#### 2.3.2 Differential scanning calorimetry (DSC)

The thermal properties of the corn starch were detected using differential scanning calorimetry (PerkinElmer Thermal Analysis, Germany). For this test, 10 mg of corn starch were placed in aluminum crucibles and were heated from room temperature (25 °C) to 100 °C in a nitrogen atmosphere at a heating rate of 10 °C/min [17].

#### 2.3.3 X-ray diffraction measurement (XRD)

X-ray diffract grams were obtained with an X-ray diffractometer (Bruker) under operating conditions as follows: The X-ray generator was operated at 40 kV and 30 mA, and the scanning angle 2θ was set from 2° to 70° at a scanning rate of 1°/min. The samples were exposed to X-ray with a wavelength of 0.1539 nm [3].

#### 2.3.4 Scanning electron microscope (SEM)

Scanning electron microscope (SEM) was applied to study starch samples' surface morphology and microstructure at an accelerating voltage of 15.0 kV [18].

#### 2.3.5 Fourier transform infrared (FTIR)

FT-IR spectra of starch samples were recorded using a Fourier-Transform Infrared Spectroscopy (Bruker, Tensor II, Germany). For this purpose, corn starch samples (1 mg) were mixed with 100 mg of KBr and the compressed and then scanned in the wavenumber range from 400 to 4000 cm^−1^ [16].

### 2.4 Preparation of milk dessert

Milk dessert was prepared by the method of Bakshi et al. (2019) with a slight modification [19]. To make 900 g of dessert, 750 g of low-fat milk was mixed with 45 g of cream that have 30 g of fat. Then the mix was heated in a water bath until it reaches 40 °C, and then stir in 54 g of sugar and 30 g of starch powder (Control & DBD2, independently). The mixture was maintained at 60 °C for 10 min to dehydrate the solid particles while being constantly stirred. Once the product reaches a temperature of 40 °C, it was put into a cooling flask. After 1 min of stirring, 1 g of vanilla and 28 g of rose water were added, and the product was heated to 4 °C [20]. Prepared dairy desserts were evaluated in terms of humidity, pH, total soluble-solid (°BRIX), syneresis, viscosity, and sensory properties. Hedonic (7-point) test was used to examine sensory properties (flavor, texture and overall acceptability). This test was conducted by panelists and was evaluated in 7-point hedonic scale. The number 7 was considered for the best sensory properties and the number 1 was considered for the worst sensory properties. Each test was repeated three times and the average of the obtained numbers was reported.

### 2.5 Statistical analysis

Generalized linear model (GLM) linear model in the SPSS statistical software was used to study the effect of plasma treatment on the corn starch physicochemical characteristics (gel transparency and OAC) and the effect of cold plasma treated corn starch on the physicochemical characteristics (Humidity, pH, Syneresis, Brix, Viscosity, Flavor, Texture and Overall acceptability) of milk dessert. Statistical analysis was done based on a one-way analysis of variance (ANOVA). The comparison of means and significant differences between replicates was conducted through Duncan's mean tests at p < 0.05.

## 3 Results and discussion

### 3.1 Physicochemical characteristics of modified corn starch

Physicochemical characteristics of modified corn starch include gel transparency, OAC (g/g), RDS (%) SDS (%) RS (%). Table 1 shows the physicochemical (gel transparency and OAC) and digestibility properties (RDS, SDS and RS) of plasma treated corn starch. According to Table 1, there were significant differences in transparency among the starch treatments (p < 0.05). The DBD2 sample exhibited higher transparency compared to the other samples. The difference observed between the two samples DBD1 and DBD2, is due to the difference in amylose content and swollen starch grains. According to the results, holes are created on the surface of starch granules by the plasma treatment process, which can cause more amylose to come out and even cause some small granules to break down, which also increases the transparency of the gel. A study has suggested that increased light transmission percentage in treated starches is likely due to the augmentation of hydroxyl groups. This, in turn, promotes the formation of hydrogen bonds with water molecules inside the starch granules, leading to improved transparency in the starch gel [21]. According to Table 1, starches treated with cold plasma exhibited higher OAC than the control sample. The DBD2 sample demonstrating significantly higher OAC (p < 0.05). According to a reported similar work by Ashwar et al. (2016) a dual autoclaving-retrogradation treatment was used for preparing resistant starch from four rice cultivars. They confirmed that oily compounds were physically trapped within the cavities and channels of the granule structure and could also form amylose-lipid complexes. They also reported dual autoclaving-retrogradation treatment of starch resulted in significant increase in resistant starch yield, water and oil absorption capacity, transmittance, freeze thaw stability, and bile acid binding with decrease in solubility and swelling index [22].

**Table 1:** Physicochemical and digestibility properties of plasma treated corn starch.

Sample^∗^	Gel transparency	OAC (g/g)	RDS (%)	SDS (%)	RS (%)
Control	1.646 ± 0.06^c^	2.32 ± 0.01^c^	56.47 ± 1.03^a∗∗^	36.55 ± 1.06^a^	6.98 ± 0.37 ^b^
DBD1	1.688 ± 0.03^b^	2.49 ± 0.02^b^	53.07 ± 0.39 ^b^	38.02 ± 0.61^a^	8.89 ± 0.67^a^
DBD2	1.785 ± 0.04^a^	2.64 ± 0.04^a^	51.86 ± 2.14 ^b^	39.06 ± 2.93^a^	9.08 ± 0.80^a^

*Data are reported in three replications in terms of (Mean ± SD value).

** The similar small letters (a-b) in each column indicate a significant no difference (p < 0.05) based on the Duncan test.

This contributes to the entrapment of oil compounds and ultimately increases the OAC compared to the control sample. Furthermore, the rearrangement of crystalline and amorphous parts within the granule and the formation of amylose-lipid complexes can enhance the physical absorption of oil [23].

According to Table 1, natural corn starch had RDS, SDS, and RS contents of 56.47 %, 36.55 %, and 6.98 %, respectively, indicating its high digestibility. However, the application of cold plasma led to a significant increase in RS and a significant decrease in the RDS of corn starch (p < 0.05). In the DBD2 sample, the RDS, SDS, and RS contents were 51.86 %, 39.06 %, and 9.08 %, respectively. The sensitivity of starch molecules to amylase affects starch digestibility, which is related to its microstructure. The amorphous structure of starch granules influences SDS production, while RS is typically derived from structures with greater order and stability [24]. In this study, the reduction in RDS values and the increase in SDS and RS values indicate the effect of cold plasma treatment on the starch structure and the arrangement of molecular chains in corn starch. Changes in RDS, SDS, and RS suggest that the structural modifications of starch create areas for amylase activity, resulting in slower or no hydrolysis by the enzyme [25]. In present study, the DBD2 treatment exhibited higher RS and SDS than the DBD1 and the control sample, and the significant increase (p < 0.05) in RS in the DBD2 sample is likely attributed to the crosslinks induced by plasma treatment. The highly energetic electrons generated after plasma treatment can stimulate argon (Ar) and oxygen (O_2_), resulting in polarity changes of O-H bonds in some hydroxyl groups. The energy transferred by Ar or O_2_ during collisions causes the detachment of O-H groups from two different glucosyl units, leading to their connection by an ether bond (starch-o-starch). According to reports, starches become indigestible due to forming ether bonds [10].

Sun et al. (2022) have investigated the effect of cold plasma and microwaves on the physicochemical properties and digestibility of rice starch. The results of their research confirm the results of the present research to a large extent. As in this research, cold plasma has caused a decrease in RDS values and an increase in SDS and RS values in corn starch, in the research of Sun et al. (2022) also reported a similar result [26].

### 3.2 Swelling power and water solubility index

**Fig. 1:**
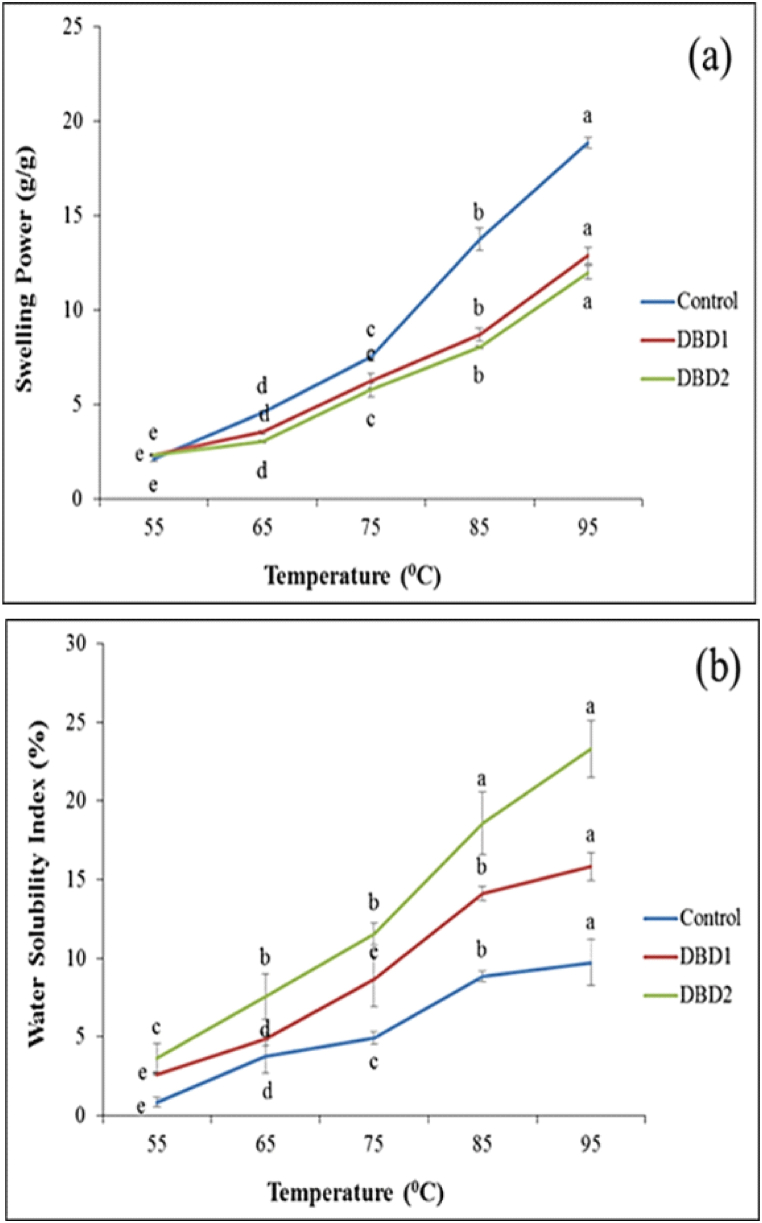
Swelling power (a) and water solubility index (b) of cold plasma treated corn starch: The similar small letters (a–e) indicate a significant no difference (p < 0.05) based on the Duncan test between the data.

Fig. 1 shows swelling power and water solubility index of corn starch treated with cold plasma. According to the results, SP and WSI significantly increased (p < 0.05) with the application of cold plasma treatment and increasing temperature from 55 to 95 °C. According to the results, the WSI of the solution increases due to the increase in polar groups at the surface after oxygen plasma application. According to Fig. 1-a, the solubility of all three starch samples increased, with the highest value observed at 90 °C. This can be attributed to the complete migration of amylose from the surface of amylopectin crystals, resulting in increased solubility. The heating process involves the involvement of amyloses in the amorphous parts of the granule in new structures, such as amylose-amylose, amylose-amylopectin, and amylose-lipid, reducing their ability to leach out of the granule [27]. The solubility increased further after cold plasma treatment, especially in the DBD2 sample. This could be due to the breakdown of amylose structures into smaller ones and the destruction of amylopectin structures, producing linear structures resembling amylose [28]. The treatment technique significantly affects the SP of starch samples (Fig. 1-b). As illustrated, there is a significant difference (p < 0.05) between the control sample and samples with plasma treatments. So, the highest and lowest values are related to the DBD2 sample and the control and DBD1 treatments, respectively. Additionally, Fig. 1-b shows that the SP of the treated corn starch, due to the thermal process, was significantly lower than that of the control sample (p < 0.05). However, as the temperature increased, the SP increased in all three starch samples. Heating the starch suspension weakens hydrogen bonds in the granule's crystalline structures, causing gradual swelling as water enters the granules. The lower SP at higher temperatures may be attributed to the rearrangement of crystalline regions, which increases the connectivity between starch chains and hinders further diffusion of amylopectin molecules [16].

In a similar research, Zuo et al. (2024), have investigated the effect of plasma treatment on corn starch. The results of their research in terms of the effect of plasma treatment on the swelling power of starch are consistent with the results of the present research. They reported that the treatment of starch with cold plasma reduces the swelling power, and a similar result was observed in this research [29].

### 3.3 X-ray diffraction and differential scanning calorimetry

**Fig. 2:**
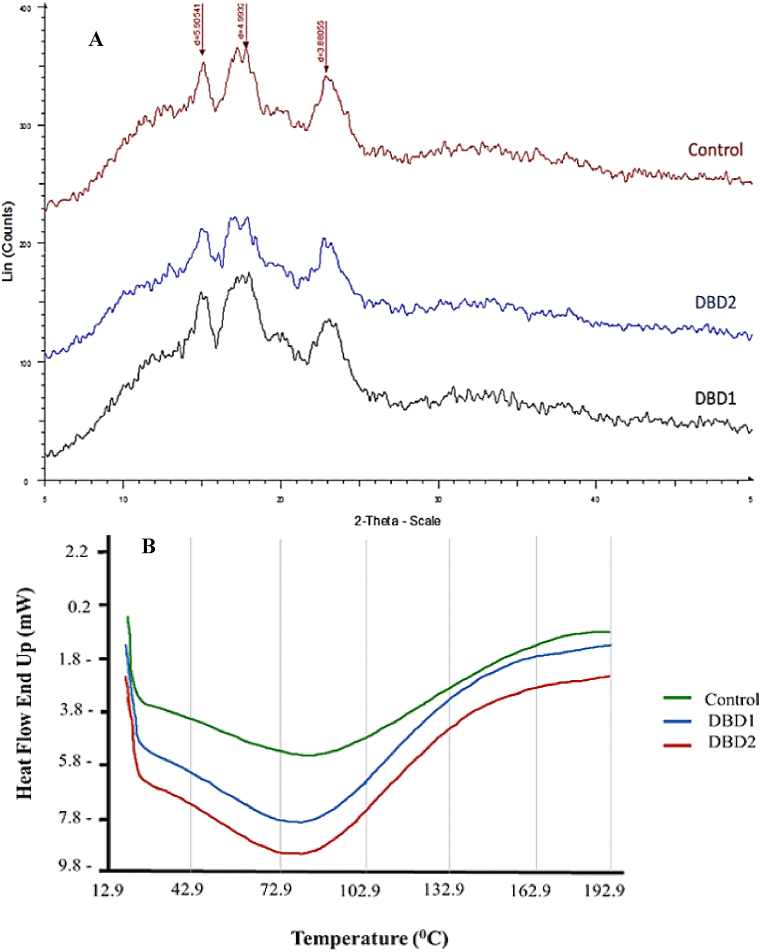
XRD (A) and DSC (B) spectra of plasma-treated corn starch.

Fig. 2 show the XRD and DSC spectra of plasma treated corn starch. XRD analysis were used to investigate the crystallinity and internal structure of corn starch samples treated with cold plasma under different argon contents (90 and 95 %) and various combinations of hydrogen and oxygen. Fig. 2-A shows the XRD patterns of Samples DBD1 and DBD2 and the control sample. Starch crystallinity is classified into A, B, C, and V types, considering the amylopectin side chain and double helixes. The main sharp and strong peaks of starch in all three samples are shown at 5.9°, 4.92°, and 3.87°. Crystallization is fairly reduced with a 10 % reduction in argon content and the replacement of hydrogen with oxygen (90 % argon gas and 10 % oxygen). The relative crystallinity decreased from 49.06 % in untreated starch to 35.47 % in the plasma treatment using 90 % argon gas and 10 % oxygen. Decreased relative crystallinity can be because of the polymerization of starch molecules induced by plasma treatment [30]. Zhang et al. (2015) reported that physical treatments mostly affect amorphous regions of starch molecules [31]. Amorphous regions of starch grains are more sensitive than crystalline regions.

Starch gelatinization occurs when amylopectin double helix molecules transition from semi-crystalline to amorphous. In analyzing the thermal characteristics, the transition temperature (T_o_, T_p_, and T_c_) indicates the extent of double helix completion, while the gelatinization enthalpy value (ΔH) represents the content of the double helix in joules per gram [16]. Fig. 2-B displays the DSC thermogram, and Table 2 presents the thermal characteristics (T_o_, T_p_, T_c_, and ΔH) of corn starch samples treated with cold plasma. Cold plasma treatment significantly reduced all four factors (p < 0.05), and the decrease in gelatinization temperature can be attributed to the polymerization of corn starch caused by cold plasma treatment. Gelatinization temperature is influenced by the degree of crystallization, the amylose-to-amylopectin ratio, and water content, so the reduction in gelatinization temperature may be due to a decrease in starch crystallization in the plasma-treated starches [3]. DBD1 and DBD2 corn starches decreased gelatinization enthalpy after plasma treatment, potentially due to plasma-generated active molecular species weakening the crosslinks in amylopectin side chains [32]. The decrease in ΔH indicates lower energy requirements for starch gelatinization. Several studies have reported changes in thermal characteristics resulting from cold plasma treatment. For instance, Okyere et al. (2019) found that cold plasma treatment of waxy starch decreased gelatinization temperature and increased ΔH, attributing the temperature reduction to starch granule polymerization and the increase in ΔH to the formation of strong crosslinks in amylopectin chains [10]. Another study on the application of dielectric plasma to corn starch reported increased gelatinization temperature and decreased starch enthalpy, suggesting the destruction of structural areas within corn starch granules [29].

**Table 2:** Thermal properties of the control and modified starch samples.

Sample	T_o_ (°Ϲ)	T_p_ (°Ϲ)	T_c_ (°Ϲ)	ΔH (J/g)
Control	41.74 ± 0.55^a^	89.33 ± 0.25^a^	139.50 ± 0.84^a^	28.15 ± 0.17^a^
DBD1	45.61 ± 0.69 ^b^	76.72 ± 0.39 ^b^	154.44 ± 0.80 ^b^	26.11 ± 0.79 ^b^
DBD2	45.67 ± 0.76 ^b^	74.01 ± 0.32^c^	151.62 ± 0.65^c^	25.65 ± 0.76 ^b^

*Data are reported in three replications in terms of (Mean ± SD value).

** The similar small letters [26] in each column indicate a significant no difference (p < 0.05) based on the Duncan test between the data.

***To = onset gelatinization temperature; Tp = peak gelatinization temperature; Tc = conclusion gelatinization temperature; ΔH = enthalpy.

### 3.4 SEM and FTIR

**Fig. 3:**
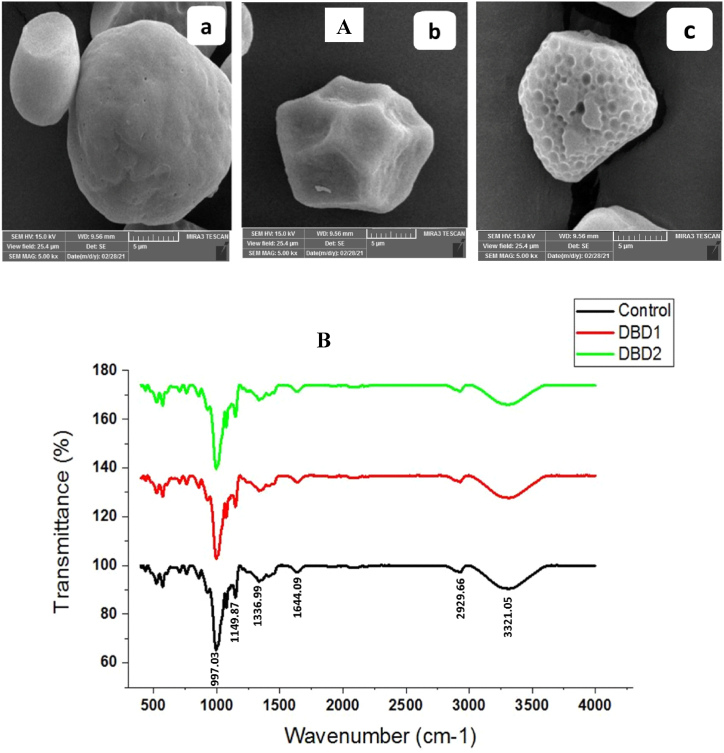
SEM images (A) (a: control; b: DBD1; and c: DBD2) and FTIR spectra (B) of cold plasma treated corn starch.

Fig. 3 shows SEM images and FTIR spectra of corn starch before and after plasma treatment. More accurate micrographs of starch granule surfaces are obtained using SEM images. The micrographs show that the starch granules have irregular, hexagonal, and polygonal shapes containing very few holes on their surface. Therefore, the types and sizes of corn starch granules remain almost similar. According to the SEM images, DBD2 sample has more and deeper fissures on its surface compared to DBD1 sample. According to the results, DBD2 sample or DBD1 treated using cold plasma under 90 % argon gas and 10 % oxygen gas had the best characteristics compared to the control sample and the plasma-treated sample under other gas contents. This can be due to the effect of plasma reactive species creating the fissures on the surface of the granules, hence, the plasma ions penetration to the molecular level and resulting in the cross-linking of the starch granules [10]. Also, the increased values may be a result of plasma treatment removing some branches in amylopectin, which could result in longer internal chain segments reacting with iodine [33], and may be a result of polymerization of the amylopectin molecule into shorter chain fragments [34].

The results of examining the chemical groups in the treated corn starch samples compared to the control sample (Fig. 3-B) show that both types of cold plasma treatment do not change the chemical groups. In the FTIR spectrum, a long peak with a wavenumber of 997 cm^−1^ was observed, and there was no significant change in DBD1 and DBD2 samples due to cold plasma application; also, no changes in their intensity were observed in other wavenumbers. The FT-IR spectrum pattern of corn starch did not change significantly after the applied treatments, which indicated no change in the chemical groups of corn starch. It was observed in several studies that the application of cold plasma on different starches did not significantly change their chemical groups, which is in line with the present study. Among these studies, it is possible to point out the effects of plasma on the non-change of chemical groups and the appearance of new peaks in rice starch [16], and also in red adzuki bean starch [35]. In the FTIR spectrum, a long peak with a wave number of 997 cm^−1^ was observed in all three starch samples, which is related to the intramolecular hydrogen bonding of hydroxyl groups at C-6 of starch. In the study of Sun et al. (2022), it was reported that the absorption intensity at 994 cm^−1^ of rice starch decreased after plasma treatment, which the researchers attributed to the reduction of intramolecular hydrogen bonds of hydroxyl groups in C-6 due to the applied treatments [35]. According to studies, it has been proven that the change in the properties of the crystalline and amorphous regions of starch can be obtained by determining the absorption ratio of 1047 cm^−1^ to 1022 cm^−1^ wavenumbers; accordingly, the ratio of 1022/1047 cm^−1^ in the present study was obtained for three control samples, DBD1 and DBD2, about 1.12, based on the similar amount in all three samples, it can be stated that corn starch has not changed in terms of crystal properties due to treatment with cold plasma. In the present study, the change in the absorption ratio of the bands at 1047/1022 cm^−1^ was not consistent with the crystallinity trend measured by XRD, which is probably because FT-IR can only penetrate the surface of starch granules, not the whole starch granules [35].

### 3.5 Physicochemical characteristics of milk dessert

Physicochemical characteristics of milk desert include humidity, pH, syneresis, °brix, viscosity, flavor, texture and overall acceptability. Table 3 shows that the effect of plasma treated corn starch on milk dessert moisture and pH variations are significant (p < 0.05). The sample treated with plasma has high pH values, this is probably due to the formation of carboxyl and peroxide groups during the plasma process, which was created during the oxidation of starch grains during plasma treatment with oxygen gas and caused a decrease in the pH and an increase in the acidity of the final product [36]. The effect of treatment on total soluble-solid (°BRIX) variations is also meaningful (p < 0.05). In this research, the plasma-treated sample contains a lower percentage of water-soluble solids. Plasma starch bombardment using high-energy ions causes depolymerization in the amylose and amylopectin side chains in the starch molecule, leading to smaller fragments [37]. Thus, the starch normally produces maltose, maltotriose, and maltotetraose after depolymerization, which in turn changes the percentage of water-soluble solids or °BRIX, and improves the properties of starch [38]. According to Table 3, the effect of plasma treatment on the syneresis changes is insignificant (p > 0.05). Plasma treatment mainly leads to the degradation and discontinuation of polymer chains under treatment. This leads to the formation of chains with different radical and mobile tails, hence, rearrangement of the polymer chain and changes in the crystallization of the polymer by forming different bonds and chains. On the other hand, most studies indicated that plasma treatment with oxygen, air, and carbon dioxide has no or minor effect on the permeability of polymers into water vapor and oxygen. However, it was concluded that a decrease in water vapor permeability after plasma treatment is due to the plasmatic conditions [38]. Viscosity refers to the resistance of an object or material against movement (flow) on a vertical or parallel plane. According to situations, the plasma process can modify the total free energy of the polymer surfaces to increase adhesion and even non-dispersion properties conditions [38]. Wu et al. (2019) studied the effects of atmospheric pressure plasma jet treatment on corn starch for 30 min at different power levels (400–800 W). A decrease in adhesion properties, final viscosity, and backwardness of starch samples (up to 1.87 %, 92.7 %, and 93.3 %, respectively) was observed by increasing plasma intensity [39].

**Table 3:** Physicochemical properties of milk dessert.

Parameters	Dessert sample	
	Control	DBD2
Humidity (%)	75.01 0.13^b^	78.04 0.07^a^
pH	6 0.07^b^	6.52 0.06^a^
Syneresis	0.05 0.03^a^	0.04 0.03^a^
°Brix	18.01 0.04^a^	14.03 0.1^b^
Viscosity (Pa. s)	26.31 0.06^a^	25.12 0.05^b^
Flavor	6.5 0.2^b^	6.8 0.3^a^
Texture	6.35 0.3^b^	6.8 0.5^a^
Overall acceptability	6.3 0.5^b^	6.75 0.5^a^

Data are reported in three replications in terms of (Mean ± SD value).

The similar small letters in each row indicate a significant no difference (p < 0.05) based on the T-test between the data.

According to Table 3, the effect of treatment on sensory properties (flavor, texture and overall acceptability) was significant (p < 0.05). The milk dessert sample prepared with modified starch has a smooth, transparent surface and a uniform texture compared to the sample prepared with Nash treatment starch. Plasma treatment can cause reactions such as starch polymerization, starch cross-linking, changes in the crystal structure of starch and its antioxidant activity [40] that affect other parameters such as taste and aroma, color and overall acceptance of food products. As a result, might significant difference has been observed in some of organoleptic properties. A study investigated modified starches in milk puddings, highlighting that corn starch contributes to normal consistency and texture. However, incorporating modified corn starch in puddings and dairy desserts results in a smooth surface, increased product stability, and resistance to temperature changes. Starch modification can also enhance viscosity control, gel strength, appearance, and synergism, making modified starches suitable for producing puddings and dairy desserts [41].

## 4 Conclusion

This study reports the application of cold plasma treatment with two different gas combinations (95 % argon+5 % hydrogen (DBD1) and 90 % argon+10 % oxygen (DBD2)), to corn starch for 10 min. The investigation demonstrated that cold plasma treatment significantly increased the production of RS. Additionally, modified starches exhibited higher transparency, WSI, and OAC than regular corn starch. However, plasma treatment reduced SP, gelatinization temperature, enthalpy, and degree of crystallization of the starch samples. In this study, DBD2 corn starch was utilized to produce modified corn starch for a dairy dessert food model. The investigation of the dairy dessert revealed that the plasma process affected pH, Brix, and viscosity, resulting in the final product with the highest pH, and low Brix and viscosity. As a chemical-free and environmentally compatible technology, plasma treatment holds great potential for producing modified starches in the future.

## Ethics statement of sensory evaluation

The author(s) confirm that the appropriate protocols for protecting the rights and privacy of all participants were utilized during the execution of the research, e.g. no coercion to participate, full disclosure of study requirements and risks, written or verbal consent of participants, no release of participant data without their knowledge, ability to withdraw from the study at any time.

## Funding declaration

The author(s) received no financial support for the research, authorship, and/or publication of this article.

## Data availability statement

Data will be made available on request.

## Consent for publication

Not applicable.

## CRediT authorship contribution statement

**Hannaneh Bahmanpour:** Resources, Formal analysis, Data curation. **Narmela Asefi:** Supervision, Software, Project administration. **Aynaz Alizadeh:** Validation, Methodology. **Sajad Pirsa:** Writing – review & editing, Validation.

## Declaration of competing interest

The authors declare that they have no known competing financial interests or personal relationships that could have appeared to influence the work reported in this paper.
